# Neurodegenerative disease and antioxidant biomarkers: A bidirectional Mendelian randomization study

**DOI:** 10.3389/fneur.2023.1158366

**Published:** 2023-03-23

**Authors:** Qianqian Zhang, Qingyang Li, Huihui Zhao, Mingzhu Shu, Maotao Luo, Yanan Li, Yu Ding, Shiyu Shi, Xi Cheng, Qi Niu

**Affiliations:** Department of Geriatrics, The First Affiliated Hospital of Nanjing Medical University, Nanjing Medical University, Nanjing, Jiangsu, China

**Keywords:** Alzheimer’s disease, Parkinson’s disease, amyotrophic lateral sclerosis, neurodegenerative diseases, antioxidant biomarkers, bidirectional Mendelian randomization study

## Abstract

**Objective:**

Previous observational studies have suggested that antioxidant imbalance is correlated with neurodegenerative diseases, while its cause–effect remains unclear. Thus, the goal of the present study is to explore the causal relationship between 11 antioxidant biomarkers and 3 most common neurodegenerative diseases [Alzheimer’s disease (AD), Amyotrophic Lateral Sclerosis (ALS) and Parkinson’s disease (PD)].

**Methods:**

A bidirectional Mendelian randomization (MR) study was performed to investigate the causal effects by using 3 main methods (Variance Weighted (IVW), Weighted Median (WM), and MR-Egger regression) in the European population. The data of 11 antioxidant biomarkers were obtained from the open database by the most up-to-date Genome-Wide Association Studies (GWAS), the summary statistics of PD and ALS were obtained from the International Parkinson’s Disease Genomics Consortium (IPDGC) (33,674 cases, and 449,056 controls), and the International Amyotrophic Lateral Sclerosis Genomics Consortium (IALSC) (20,806 cases and 59,804 controls), respectively. For AD, we specifically used two recently published GWAS data, one from the International Genomics of Alzheimer’s Project (IGAP) (21,982 cases and 41,944 controls), and the other from a large meta-analysis (71,880 cases and 383,378 controls) as validation data.

**Results:**

Based on the Bonferroni correction *p* < 0.0015, there was no significant causal evidence for the antioxidant biomarkers on neurodegenerative diseases, however, the reverse analysis found that AD was significantly related to the decrease in retinol (IVW: beta = −0.023, *p* = 0.0007; WM: beta = −0.025, *p* = 0.0121), while the same analysis was carried out between the AD validation database and retinol, the results were consistent (IVW: beta = −0.064, *p* = 0.025). Moreover, AD on Glutathione S-transferase (GST), PD on Glutathione Peroxidase (GPX) as well as PD on uric acid (UA) also indicated potential causal-and-effect associations (IVW: *p* = 0.025; *p* = 0.027; *p* = 0.021, respectively).

**Conclusions:**

There was no sufficient evidence that antioxidant imbalance has a significant causal effect on neurodegenerative diseases. However, this study revealed that genetically predicted AD was significantly related to the decrease in retinol, which provides a new insight into previous research and indicates the possibility to regard retinol as potential biomarker for the diagnosis and progress of AD.

## 1. Introduction

The phrase “neurodegenerative disease” refers to a spectrum of disorders in which neurons lose their function and structure gradually over time, ultimately leading to neurological dysfunction (1). The incidence of neurodegenerative diseases, including Alzheimer’s disease (AD), Parkinson’s disease (PD), Amyotrophic Lateral Sclerosis (ALS), has increased globally as a result of an aging population, and it is now a major social concern in many countries. These diseases frequently share the same pathogenesis involving misfolding, aggregation, deposition of abnormal proteins, and may be connected to the aggregation of proteins with fibril formation or deposition of amyloid, even though the type of aggregated proteins, region of deposition, and cellular distribution vary with each disease (2). Despite a large number of studies on neurodegenerative diseases, the etiology and pathogenesis are still unclear. Recently, numerous studies have found that oxidative stress injury may shift the balance of proteins and result in neurodegenerative diseases (3).

Oxidative stress is the result of the imbalance between reactive oxygen species (ROS) formation and enzymatic and non-enzymatic antioxidants. Antioxidant is defined as a substance that can delay or prevent oxidative damage caused by the presence of ROS, when it exists at a low concentration compared with oxidizable compounds (4). Antioxidants function means reducing oxidative stress, DNA mutation, malignant transformation and other cell damage parameters. Antioxidants can be divided into two groups: Enzymatic antioxidants and non-enzymatic antioxidants. Enzymatic antioxidants include Glutathione S-transferase (GST), Superoxide Dismutase (SOD), Catalase (CAT), and Glutathione Peroxidase (GPX), while non-enzymatic antioxidants are Uric acid (UA), Glutathione (GSH), various vitamins (carotenoids, vitamin E, A, and C), and some transition metalions (5).

Many antioxidants such as retinol, ascorbic acid and vitamin E are also nutrients (6). Literature is rich with evidence that neurodegenerative diseases are correlated with the unbalance of these antioxidants. Studies have found that antioxidants are abnormal in patients compared with healthy controls. For example, ascorbate, vitamin A, vitamin E and GSH levels in AD and PD patients’ serum, all have varying degrees of decrease (7–9). Moreover, several experimental evidence has demonstrated that taking antioxidants is beneficial on treating neurodegenerative diseases. Research has shown that taking vitamin E supplements significantly decreased the number of ALS deaths (10). It has also been shown that omega-3 plus alpha lipoic acid supplements can slow cognitive and functional decline in AD patients over a 12-month period (11).

Even so, it is still difficult to illustrate whether neurodegenerative diseases are caused by antioxidant imbalance. Mendelian randomization (MR) is an analytical method used to evaluate the causal relationship between observed modifiable exposure or risk factors and clinically relevant results. It serves as a valuable tool, especially when randomized controlled trials are not feasible to examine the causation and observational studies provide biased associations due to confounding or reverse causal relationship (12). However, when the precise biological function of a person’s genetic variation used in an MR is unknown, conventional MR analysis is likely to produce erroneous conclusions about the direction of causation. Bidirectional MR can quantify the effect of each variable on causality and minimize the occurrence of such errors (13). Therefore, we chose 11 common antioxidant biomarkers to establish the causal relationship between antioxidant imbalance and neurodegenerative diseases by conducting a bidirectional Mendelian randomization study.

## 2. Materials

### 2.1. Study design

**Figure 1:**
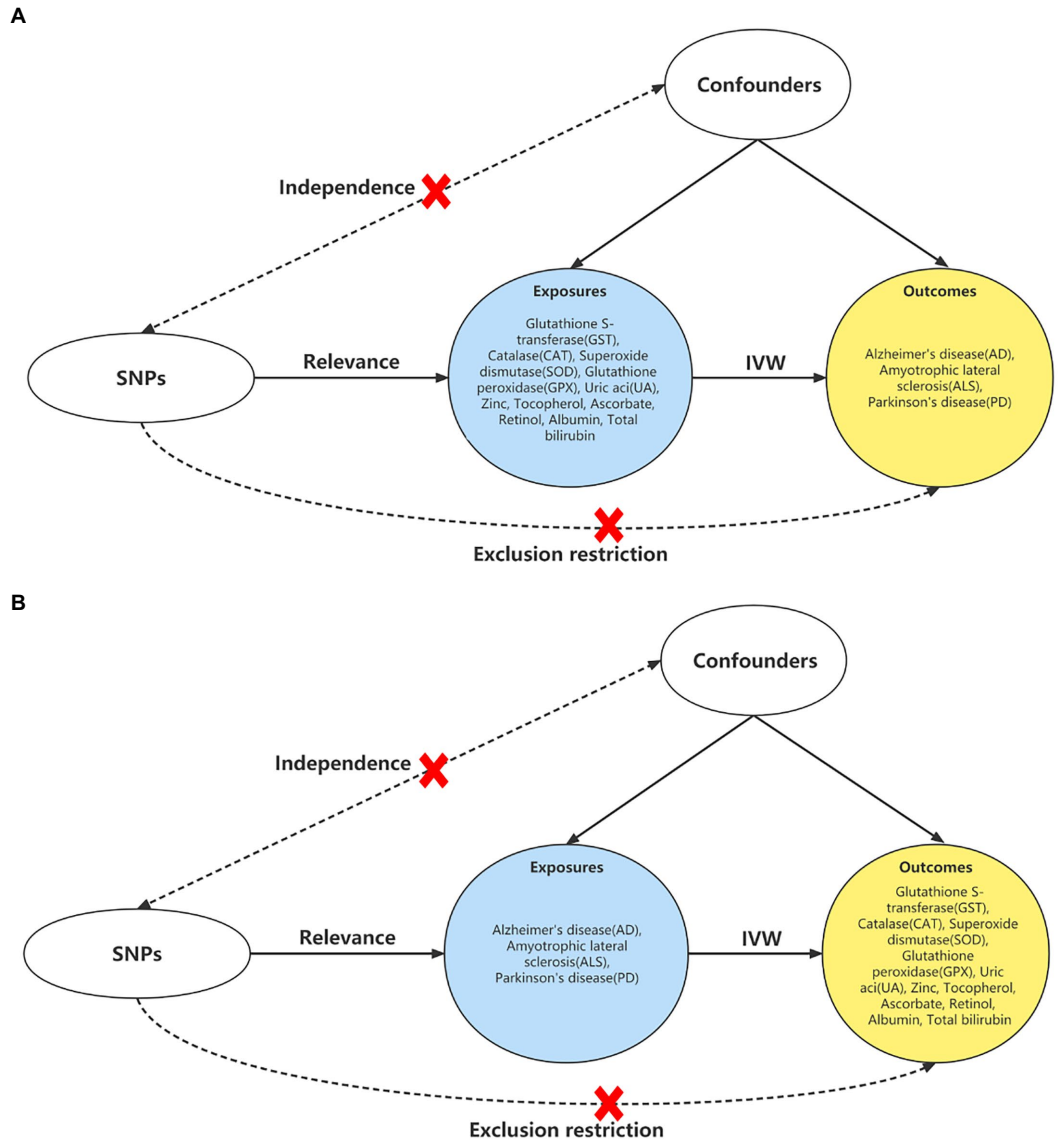
Study design of the bidirectional MR. **(A)** Study design of the causal effect of antioxidant biomarkers on the neurodegenerative diseases. **(B)** Study design of the associations of neurodegenerative diseases on antioxidant biomarkers. Abbreviations: MR: Mendelian randomization; IVW, inverse variance weighted method; SNP, single nucleotide polymorphism.

All the summarized data analyzed were accepted only from the European population in order to reduce genetic heterogeneity. We included 11 biomarkers of antioxidants (14) from the open database by the most up-to-date Genome-Wide Association Studies (GWAS) (accessed on 1 November 2022)^1^ utilizing a bidirectional MR study to investigate the causal effects on 3 neurodegenerative diseases (AD, ALS, and PD) (Figure 1). The MR approach indicates that three assumptions are required: Firstly, the Single Nucleotide Polymorphisms (SNPs) that served as Instrumental Variables (IVs) in MR were derived from GWAS and are associated with exposures; next, IVs are not related to the confounders; finally, IVs do not affect the outcome other than through the exposure (15).

### 2.2. Data sources

For the antioxidant biomarkers, summary statistics of CAT, SOD, GST, and GPX were selected from the study of INTERVAL (3,301 individuals) (16), tocopherol (6,266 individuals) and albumin (115,060 individuals) were obtained from the Twins UK cohort and the KORA study (17), while the data of Zinc including 2,630 individuals was derived from the MR-Base project which was generated by a variety of consortia (18); finally, the ascorbate (64,979 individuals), UA (343,836 individuals), retinol (62,911 individuals), and total bilirubin (TBIL) (342,829 individuals) data were selected from the UK biobank.

For the neurodegenerative diseases, the ALS database was obtained from the International Amyotrophic Lateral Sclerosis Genomics Consortium (IALSC) (20,806 cases and 59,804 controls) (19). The EI Escorial criteria were used for diagnosing patients with probable or definite ALS (20). In addition, a recent PD GWAS meta-analysis from the International Parkinson’s Disease Genomics Consortium (IPDGC) including 3 previously reported GWAS studies, 13 new datasets, as well as UKB proxy-case data (excluding 23andMe) was used as the PD source (33,674 cases, and 449,056 controls) (21). For AD, we used two recently published GWAS data, one from an International Genomics of Alzheimer’s Project (IGAP) meta-analysis of stage 1 as AD data in which 63,926 individuals were included (21,982 cases and 41,944 controls) from four consortiums as primary data for analysis (22), and the other from a large meta-analysis (71,880 cases and 383,378 controls) which was used as validation cohort.

The MungeSumstats package (version 1.6.0) was used to process all GWAS summary statistics in R (version 4.2.2) for quality control and to generate reformatted data objects with standardized columns for downstream analysis (23). A detailed description of the GWAS datasets can be found in Supplementary Table S1 informed consent and ethical approvals were got for original studies. All data were available and openly accessible to the public.

### 2.3. Genetic IVs

More than 10 independent SNPs as IVs are recommended for MR analyses to keep statistics at a sufficient level (24), and the SNPs should be independent of each other. We set the GWAS value of *p* < 1 × 10–5, *r*^2^ < 0.01, and distance >250 kb to get enough SNPs and exclude variants in strong Linkage Disequilibrium (LD) (14). Furthermore, during the harmonization process (25), we excluded palindromic SNPs and the SNPs close associated with the outcomes (*p* < 1 × 10–5). Due to the second assumption of the MR approach, we also searched for potential pleiotropy in the PhenoScanner v2 database (*p* = 1 × 10–5).^2^ The SNPs would be specifically removed for the analysis in Figure 1A if related to hypertension, diabetes, smoking, obesity, depression, cholesterol, and hard of hearing on AD (26), head trauma, intake of antioxidants, and smoking on ALS (27, 28), caffeine consumption on PD (29). For Figure 1B, we would filtered the SNPs of acute lymphocytic leukemia (30), Crohn’ s disease (31), rheumatoid disease (32), asthma (33), supplements of vitamin B complex (34), smoking (35), and ulcerative colitis (36), which were reported to have a potential impact on the antioxidant biomarkers. Finally, we calculated the *F* statistics before MR analysis, and all SNPs were greater than 10, suggesting the strong instruments of IVs (37). The detailed information of the SNPs for each outcome are displayed in Supplementary Table S2.

### 2.4. Statistical analysis

In this MR analysis, three methods were mainly used to identify the effects of exposures on outcomes: Variance Weighted (IVW) as the primary analysis, Weighted Median (WM) as the secondary analysis, and MR-Egger regression as sensitivity analysis. When all SNPs selected are valid IVs, the weighted average of Wald ratio estimates for each variant is calculated using the IVW method, which can provide the most accurate estimates (38). Contrary to IVW, a minimum of 50% of the weight of valid IVs are required for the WM method (39). In the MR-Egger regression method, weighted linear regression is performed based on the assumption that the direct effects of exposure are not influencing the associations between genetic variants and exposure (40). Furthermore, residual horizontal pleiotropy was investigated using the intercept test for MR-Egger regression (value of *p* < 0.05 suggesting pleiotropy). The heterogeneity of SNPs was also assessed using Cochran’s *Q* test, and the sensitivity was tested using leave-one-out sensitivity analysis. The data were corrected by removing outlier SNPs after conducting MR Pleiotropy RESidual Sum and Outlier (MR-PRESSO) to test for the presence of horizontal pleiotropy (41).

All statistical analyses were done by R software (v. 4.2.2) with TwoSampleMR (v.0.5.6) (18) and MR-PRESSO (v.1.0) (41) packages. Multiple comparisons were done by Bonferroni correction (*p* < 0.05/33) that was regarded as evidence of statistical significance. However, *p* < 0.05 was assumed suggestive evidence of a causal relationship.

## 3. Results

### 3.1. Causal role of antioxidant biomarkers on neurodegenerative diseases

**Figure 2:**
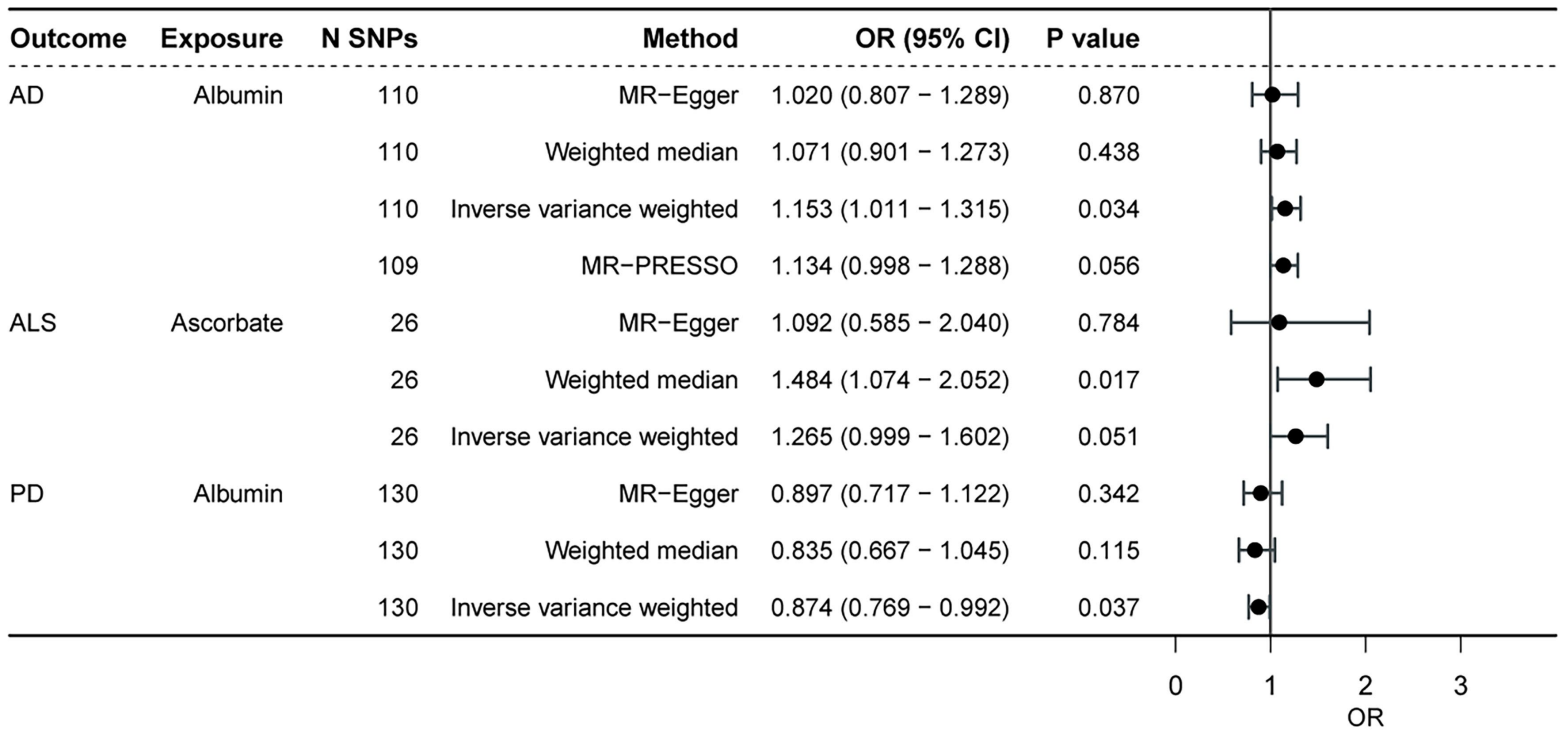
The causal effect of antioxidant biomarkers on the neurodegenerative diseases. AD, Alzheimer’ s disease; ALS, amyotrophic lateral sclerosis; PD, Parkinson’ s disease; SNP, single nucleotide polymorphism.

Based on Bonferroni correction in this section, antioxidant biomarkers did not significantly contribute to neurodegenerative diseases (with or without adjustment for potential confounders, *p* < 0.0015). However, after MR-PRESSO correction, we found that some biomarkers showed a nominal association with ALS, PD, but not AD after MR-PRESSO corrected (Figure 2). In particular, genetically predicted albumin was obviously associated with lower chances of PD (OR = 0.874, *p* = 0.037), and higher chances of AD in IVW analysis (OR = 1.153, *p* = 0.034). However, after 1 outlier SNP was eliminated by MR-PRESSO, the statistical significance of albumin on AD diminished (OR = 1.134, *p* = 0.056). Furthermore, genetically predicted ascorbate was associated with higher chances of ALS in WM analysis (WM, OR = 1.484, *p* = 0.017), and IVW analysis also showed the same trend (OR = 1.265, *p* = 0.051). Apart from the aforementioned, the MR-PRESSO test also identified 3 outliers SNPs of AD, 2 outliers of ALS, and 1 outlier. There was no difference in significance or magnitude of these associations after correcting for possible outliers (OR = 1.054, *p* = 0.098 for CAT on AD; OR = 1.020, *p* = 0.396 for GPX on AD; OR = 1.000, *p* = 0.952 for UA on AD; OR = 0.999, *p* = 0.064 for UA on ALS; and OR = 1.001,*p* = 0.819 for albumin on ALS; and OR = 1.000, *p* = 0.638 for UA on PD). Except for albumin and total bilirubin on ALS (*p* = 0.018, *p* = 0.035, respectively), there was no pleiotropy by using the MR-Egger intercept. Whether or not the potential confounders were removed, the MR analysis of the 2 showed no significant statistical differences. Supplementary Table S3 show the other detailed results.

### 3.2. Causal effect of neurodegenerative diseases on antioxidant biomarkers

**Figure 3:**
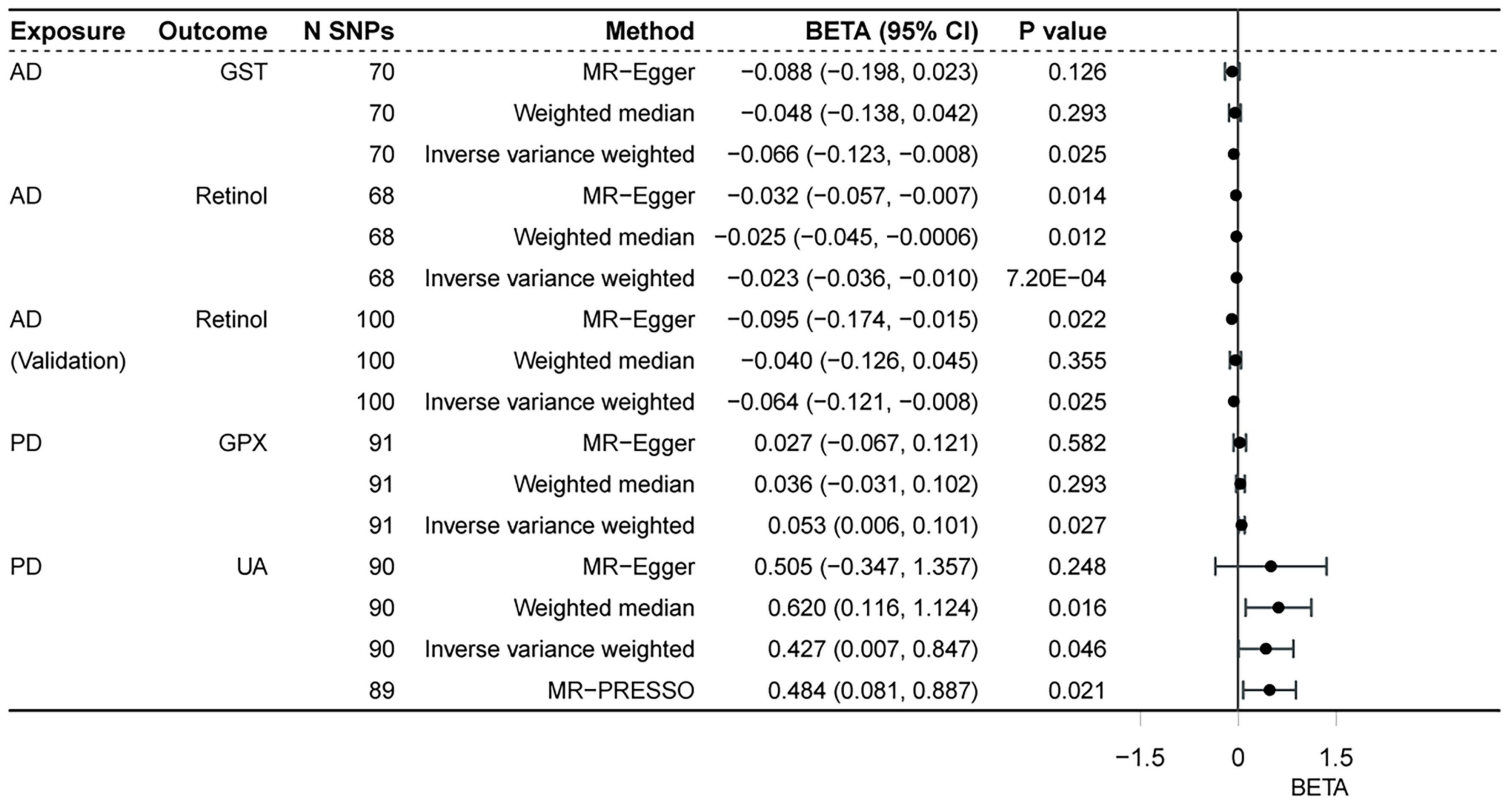
Associations of neurodegenerative disorders and antioxidant biomarkers. AD, Alzheimer’ s disease; PD, Parkinson’ s disease; GST, glutathione S-transferase; GPX, glutathione peroxidase; UA, uric acid.

**Figure 4:**
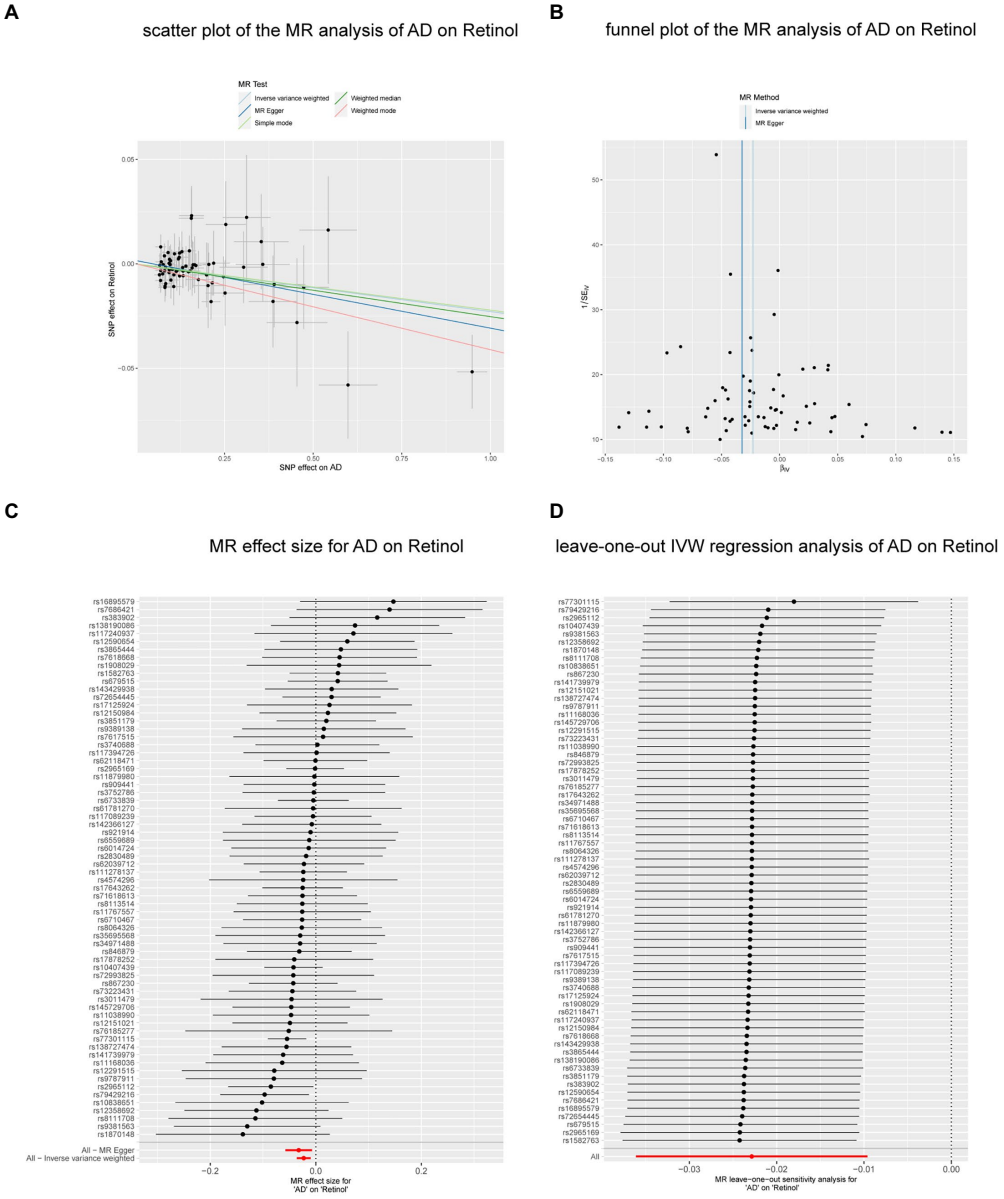
The causal effect of AD on retinol. **(A)** Scatter plot of the MR analysis of AD on retinol. **(B)** Funnel plot of the MR analysis of AD on retinol. **(C)** MR effect size for AD on retinol. **(D)** Leave-on -out IVW regression analysis of AD on retinol. Abbreviations: MR: Mendelian randomization; AD, Alzheimer’ s disease; IVW, inverse variance weighted.

We performed reverse MR analysis to evaluate the potential causal effects of neurodegenerative diseases on biomarkers associated with antioxidants. All MR-Egger intercept tests revealed no pleiotropy. In IVW analysis, AD on retinol showed a significant causal effect (*p* = 0.0007) after Bonferroni correction (*p* < 0.0015) for multiple tests, maintaining consistency with the WM method (*p* = 0.0121) and MR-Egger method (*p* = 0.0139, *p* of egger intercept = 0.386). The findings of Cochran’s *Q* test (*p* of *Q* = 0.983) (Figure 3) as well as the sensitivity analysis of leave-one-out (Figure 4) were all consistent. Additionally, the same analysis was carried out between the AD validation database and retinol, and the results were consistent (IVW: beta = −0.064, *p* = 0.025). The Supplementary Table S4 contain detailed results.

**Figure 5:**
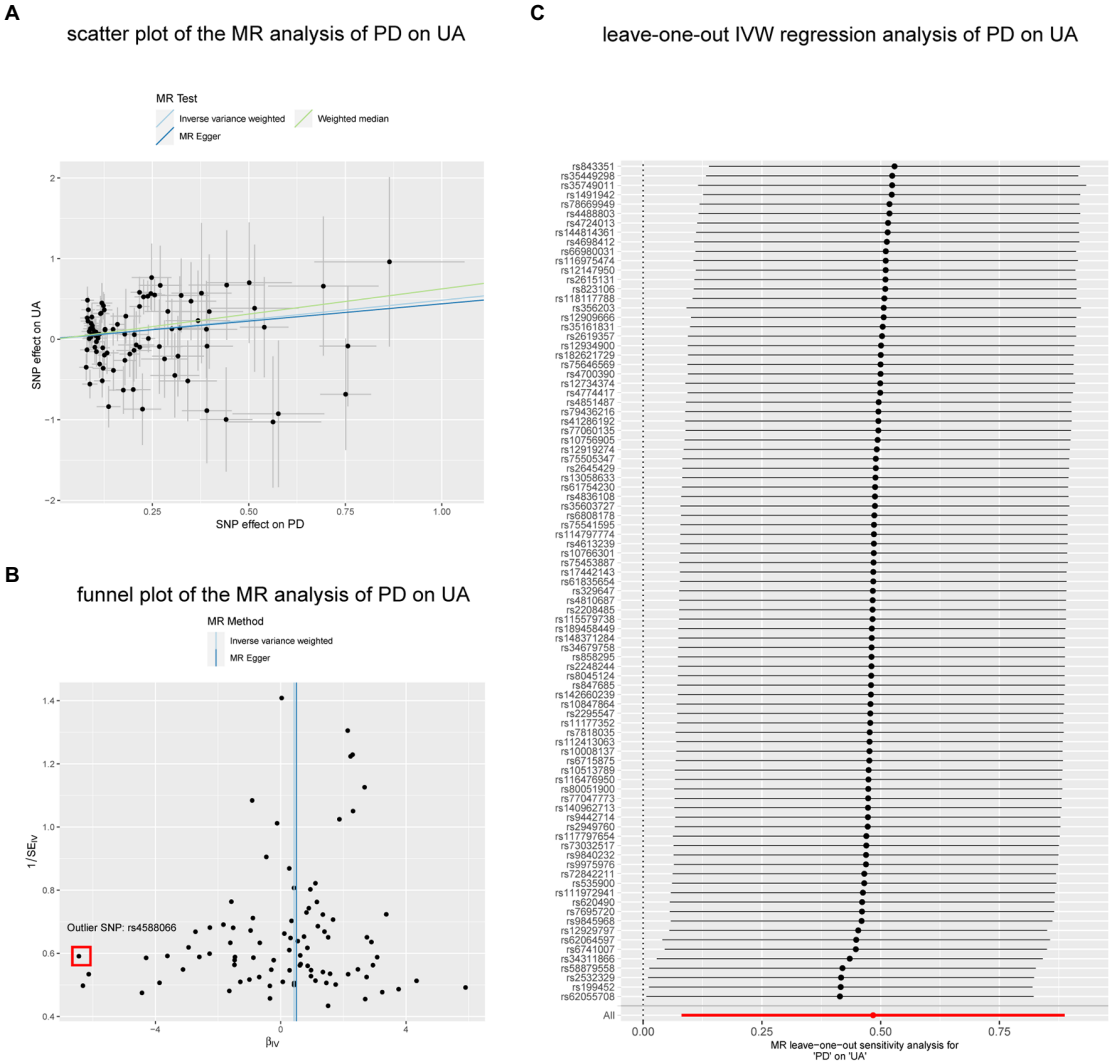
The nominal associations between PD and UA. **(A)** Scatter plot of the MR analysis of PD on UA. **(B)** Funnel plot of the MR analysis of PD on UA. **(C)** Leave-on -out IVW regression analysis of PD on UA. Abbreviations: PD, Parkinson’ s disease; UA, uric acid; IVW, inverse variance weighted method; SNP, single nucleotide polymorphism.

In the reverse analyses, we also found nominal associations between AD and GST (IVW, *p* = 0.025; *p* of *Q* = 0.976; *p* of egger intercept = 0.653), PD and GPX (IVW, *p* = 0.027; *p* of *Q* = 0.154; *p* of egger intercept = 0.519), while the relationship disappeared in the WM method (both of the two *p* = 0.293). In contrast to the previous, PD was found to be nominally associated with increased UA both in IVW (*p* = 0.021; *p* of *Q* = 2.1 × 10–7; *p* of egger intercept = 0.837) and WM methods (*p* = 0.016). The result remained consistent (MR-PRESSO, *p* = 0.021) (Figure 5) after excluding 1 outlier SNP (rs4588066).

Finally, the MR-PRESSO test detected 3 other outlier SNPs of PD on albumin (2 SNPs) and total bilirubin (1 SNP), 2 outlier SNPs of AD on total bilirubin, and 1 outlier SNP of ALS on total bilirubin, and this association’s significance and magnitude did not change after removing the outliers. Other results are available in the Supplementary Table S4.

## 4. Discussion

We performed a bidirectional MR analysis using the latest GWAS summary-level data publicly available to date to assess the causal relationship between three neurodegenerative diseases (AD, ALS, and PD) and eleven antioxidant biomarkers. Results showed that genetically predicted AD was significantly related to the decreased retinol, as well as the result of validation study, and there was also evidence of a possible causal relationship between albumin and lower chances of PD, ascorbate and higher odds ALS, AD and decreased GST, PD and increased GPX as well as UA.

Oxidative stress is caused by a redox system imbalance. The brain is particularly highly vulnerable because it has an abundance of peroxidation susceptible lipid cells that are vulnerable to oxidation and needs to a high oxygen level (42). Antioxidants are crucial in preventing oxidative stress injury. They can control the level of oxidative stress and form minor reactive species via radicals, protecting the cell from being damaged (43, 44). Numerous observational researches have shown a strong association between neurodegenerative diseases and antioxidant imbalance, especially in AD, PD, and ALS. However, very few studies have shown conclusive evidence for this association. Based on Bonferroni correction, antioxidant biomarkers in our study did not significantly contribute to neurodegenerative diseases, but there were some notional correlations between ascorbate and ALS, and between albumin with PD. In the conclusion, these associations can highlight the possibility that changes of antioxdiants have a significant role on neurodegenerative diseases. Moreover, it can be supported by clinical evidence. Currently, some clinical medicine like edaravone (MCI186-19) (45), riluzole (46), melatonin (47, 48), coenzyme Q10 (49) are widely used to treat neurodegenerative diseases. All of these medicines are associated with antioxidants. The strong relationship between antioxidants and neurodegenerative diseases must therefore be given close attention.

Enzymatic antioxidants and non-enzymatic antioxidants are the two groups into which antioxidants can be divided. Enzymatic antioxidants are SOD, CAT, GST, and GPx. These single enzymes served as the “first line of defense” to inhibit oxidative stress, prevent the production of ROS, and block the generation of free radicals. This study illustrated that AD was associated with GST as well as PD was related to GPx, which are correlated with former research (50, 51). While, uric acid, lipoic acid, bilirubin, vitamins E, A, and C, and albumin are non-enzymatic antioxidants, part of the non-enzymatic antioxidants can also be a nutrient.

Uric acid (UA), an end product from purine, which is one of the most important non-enzymatic antioxidants in the human serum. It functions as a special free radical inhibitor and can chelate metal ions like iron and copper besides acting as a radical scavenger (52). Previous studies have shown a significant decrease in serum UA in PD (53),but these observational studies have many limitations in clearly stating the causal relationship between UA and PD. Recently, minority studies had used Mendelian randomization, although it is not clear whether there is a causal link between the two. However, these results have called into question the widely held belief that low UA contributes to PD, which may help to explain the finding in our study that PD is nominally associated with increased UA (54–56).

Retinol, also known as Vitamin A, acts as an antioxidant, which plays a role in maintaining higher functions of the central nervous system. Studies have shown that lack of retinol might result in a decrease in cognitive function (8), and supplement retinol appropriately could be key molecules for the prevention and therapy of AD (57). Meanwhile, previous observable researches results have suggested that compared with healthy controls, retinol was confirmed to be lower in AD patients’ serum and plasma (58, 59). In our study, although it is difficult to prove that whether retinol supplementation improves the symptoms of AD, we found that AD was significantly correlated with the decreased retinol. Therefore, it is reasonable to illustrate that retinol can be used as a potential biomarker for AD to help early diagnosis and monitor disease progression.

Ascorbate, also called Vitamin C, has been known to have the ability to neutralize superoxide radicals (44, 60). As an antioxidant, ascorbate has a reasonable mechanism to influence the process of neurodegenerative diseases. Two preclinical studies showed benefits of ascorbate in a familial ALS mouse model, and there are two case reports indicated that ascorbate was associated with improvement of the disease. However, due to the small sample size, the lack of randomization or blinding, and the use of treatment methods before symptoms appear, these methods have defects. What’s more, these studies have never been independently replicated (61). Recently, an increasing number of studies have showed that vitamin C intake did not have any association with ALS progression. A prospective trial of vitamin C in ALS indicated that the presence of ascorbate will cause the progress of the disease and the use of ascorbate could have unfavorable effects in ALS patients (62). Our study found that ascorbate was related with higher chances of ALS by using MR study, which was consistent with some previous research results. The result was helpful to trigger further research on ascorbic acid.

Albumin, as the most abundant protein, has many properties including antioxidant and anti-inflammatory activity. Moreover, albumin has tied up with many neurodegenerative diseases because of its direct protective action on neuron (63). In our study, we also proved that it was related to PD.

We were able to demonstrate the following benefits from our study. First, the causal effect was investigated for the first time antioxidant biomarkers on neurodegenerative diseases using bidirectional MR design. Then, to partly reduce the bias, we selected the most up-to-date GWAS data and included 11 antioxidant biomarkers for the MR analysis. However, our study still has some limitations. Firstly, to include more SNPs, we set the value of p as 1 × 10–5. This implied that it will be comparatively small when it comes to explaining the percentage of variance between IVs and some exposures. The occurrence of some unidentified antioxidant biomarkers and other unavoidable undiscovered confounders may impair the causal inference even though we excluded some SNPs associated with confounders. Besides, enzymatic antioxidants are also known as intracellular antioxidants, which are primarily responsible for intracellular defence. Although it is ease to collect the samples, the enzymatic antioxidants are more extensively diluted in the blood. In terms of this issue, there are some bias in the results of the study. Moreover, we have to admit that biomarkers of antioxidant have no pathogenic effect in neurodegenerative diseases, and the result may also be due to limited statistical ability, therefore we recommend interpreting the results cautiously. Due to the limitations of MR, the lack of associations must be interpreted carefully. Finally, SOD1 was found related to ALS, especially in the people of Asian region. The study was limited to people of European origin and may not apply to other populations, which may explain the reason why we did not find a causal association between SOD1 and ALS.

## 5. Conclusion

This bidirectional MR study illustrates that antioxidant imbalance did not show definite causal effect on neurodegenerative illnesses and that AD was significantly associated with the decline in retinol. This study provides a more solid scientific basis for the previous observational studies, and reveals the significance of retinol for diagnosis and monitoring the process on AD. In conclusion, our study contributes to a deeper understanding of the relationship between neurodegenerative disorders and antioxidants and will facilitate further exploration on the treatment of neurodegenerative illnesses.

## Data availability statement

The original contributions presented in the study are included in the article/Supplementary material, further inquiries can be directed to the corresponding authors.

## Ethics statement

Ethical review and approval was not required for the study on human participants in accordance with the local legislation and institutional requirements. Written informed consent for participation was not required for this study in accordance with the national legislation and the institutional requirements.

## Author contributions

QZ and QL: designed the project, analyzed the data, and drafted the initial version of the manuscript. HZ and MS were involved in the acquisition of data. ML, YL, YD, and SS wrote some part of the manuscript. XC revised the manuscript. QN edited the manuscript grammatically and supervised the study. All authors contributed to the article and approved the submitted version.

## Funding

This work was financially supported by the National Natural Science Foundation of China (82071434), the Natural Science Foundation of Jiangsu Province (BK20201490), Nanjing Medical University Specific Disease Cohort Study Project (JX218GSP20211804A), Jiangsu Province “Six Talent Peak” High-Level Talent Selection and Training Programme (WSN-004), the 511 Project, and Jiangsu Province Hospital (the First Affiliated Hospital with Nanjing Medical University) Clinical Capacity Enhancement Project.

## Conflict of interest

The authors declare that the research was conducted in the absence of any commercial or financial relationships that could be construed as a potential conflict of interest.

## Publisher’s note

All claims expressed in this article are solely those of the authors and do not necessarily represent those of their affiliated organizations, or those of the publisher, the editors and the reviewers. Any product that may be evaluated in this article, or claim that may be made by its manufacturer, is not guaranteed or endorsed by the publisher.

## Footnotes

^1^ https://gwas.mrcieu.ac.uk/

^2^ http://www.phenoscanner.medschl.cam.ac.uk/
